# Diagnostic accuracy of serological tests for dermatitis herpetiformis: systematic review and Bayesian meta-analysis

**DOI:** 10.1186/s13643-025-03010-y

**Published:** 2025-12-01

**Authors:** Honoria Ocagli, Giacomo Berti, Cristina Canova, Serena Szekely, Stefano Piaserico, Fabiana Zingone, Ileana Baldi

**Affiliations:** 1 Unit of Biostatistics, Epidemiology, and Public Health, Department of Cardiac, Thoracic, and Vascular Sciences, University of Padova, Via Loredan, 18, 35121 Padova, Italy; 2 Dermatology Unit, ULSS 3 Serenissima, Mestre (Venice), Italy; 3 Dermatology Unit, Department of Medicine (DIMED), University of Padua, Padua, Italy; 4 Unit of Gastroenterology, Azienda Ospedale Università Padova, Padua, Italy

**Keywords:** Dermatitis Herpetiformis, Diagnostic accuracy, Serological tests, Systematic review, Meta-analysis, Gluten-free diet

## Abstract

**Background:**

The diagnosis of Dermatitis Herpetiformis (DH) relies on both clinical and serological tests. Accurate diagnostic tools are critical for effective management. This systematic review and meta-analysis aimed to evaluate the diagnostic accuracy of serological tests for DH, focusing on the impact of gluten-free diet (GFD) status on test performance, using Direct Immunofluorescence (DIF) as the reference standard.

**Methods:**

This review follows the PRISMA-DTA guidelines. The PICO framework was used to define the review question. Databases, including PubMed, SCOPUS, EMBASE, and Web of Science were comprehensively searched until July 14, 2023. The risk of bias was assessed using QUADAS-2. Bayesian meta-analyses were conducted using the MetaBayesDTA tool.

**Results:**

A total of 25 studies were included in the systematic review, with 7 studies included in the meta-analysis. The pooled sensitivity (SE) and specificity (SP) estimates varied by test type and population characteristics, highlighting the significant impact of GFD status on test accuracy and false positivity rate. The meta-analysis on the sample that included patients with celiac disease (CeD) who had not adhered to a GFD, revealed that the ELISA for IgA- tTG (SE: 0.90, SE 95% CrI: 0.71–0.99; SP: 0.14, 95% CrI: 0.03–0.44) and IIF for IgA-EMA (SE: 0.89, 95% CrI: 0.32–0.99; SP: 0.13, 95% CrI: 0.01–0.71) demonstrated high sensitivity but low specificity. Instead, ELISA for IgA- eTG (SE: 0.46, 95% CrI: 0.05–0.91; SP: 0.60, 95% CrI: 0.14–0.95) exhibited a lower sensitivity but a higher specificity than others tests.

**Conclusions:**

Selecting appropriate diagnostic tests based on clinical context is crucial for accurate DH diagnosis. Selecting appropriate diagnostic tests based on clinical context is crucial for accurate DH diagnosis. Incorporating the GFD status of patients and combining IIF for IgA EMA and ELISA for IgA tTG tests in diagnostic algorithms can improve sensitivity and specificity, leading to better patient outcomes. Future research should focus on standardizing study designs and improving patient selection methods to enhance diagnostic accuracy.

**Trial registration:**

PROSPERO CRD42023444060.

**Supplementary Information:**

The online version contains supplementary material available at 10.1186/s13643-025-03010-y.

## Background

Dermatitis herpetiformis (DH) is a skin disease characterised by intensely pruritic papulovesicular eruptions affecting most commonly the extensor surfaces of the elbows, knees and buttocks, and it is considered the cutaneous manifestation of celiac disease [1]. It primarily affects young adults but ranges from infants to older people. DH is more common in males (2:1 ratio), except in those under 20, where females predominate. Prevalence varies by country, from 1 per million in Germany to 58.8 per 100,000 in Ireland, with familial cases occurring in 2.3–10.5% of instances [2].

The diagnosis of DH poses significant challenges, mainly due to different diagnostic methodologies. Accurate identification of DH is critical, as it guides the implementation of a gluten-free diet (GFD), which is the primary treatment for managing both DH and celiac disease (CeD) [3, 4].

Diagnosis of DH involves a combination of patient’s history, physical examination, and specific diagnostic procedures. It is recommended to assess the duration, severity, and type of skin symptoms and the presence of gastrointestinal and malabsorptive symptoms. A thorough evaluation should also include a family history of CeD and DH, and the presence of associated autoimmune diseases [1]. Diagnostic procedures are crucial, with direct immunofluorescence (DIF) examination of a perilesional skin biopsy and serum tests for tissue transglutaminase or endomysial antibodies being mandatory. Although, DIF is considered the gold standard for diagnosing DH, it is important to note that it is not a perfect standard. Granular IgA deposits co-localized with TG3 in the dermal papillae are a hallmark of DH and emphasize the critical role of DIF. These deposits often persist even in patients who adhere to a gluten-free diet, highlighting the challenges in disease monitoring and the need for complementary diagnostic approaches [4]. The sensitivity of DIF is generally between 90–95%, and its specificity ranges from 95–100% [3]. However, the accuracy of DIF can be affected by various factors, such as the quality of the biopsy, the skill of the technician, and the interpretation of the results. This variability introduces uncertainty, underscoring the need for cautious interpretation of DIF results. Histopathological analysis of lesional skin biopsy and HLA DQ2 and DQ8 typing are recommended to clarify the diagnosis in unclear cases. These recommendations ensure a comprehensive and accurate diagnosis of DH. Diagnosis of DH typically involves the detection of specific antibodies, such as IgA anti-tissue transglutaminase (tTG), anti-epidermal transglutaminase (eTG), and anti-endomysial antibodies (EMA), through an enzyme-linked immunosorbent assay (ELISA) and indirect immunofluorescence (IIF).

Despite the availability of various serological testing methods, the diagnostic accuracy of these tests can vary significantly. Previous studies have provided fragmented insights into the sensitivities and specificities of these methods, but a comprehensive synthesis and comparison are lacking.

## Methods

### Protocol and registration

The systematic review is reported following the general principles recommended in the Preferred Reporting Items for a Systematic Review and Meta-analysis of Diagnostic Test Accuracy Studies: The PRISMA-DTA Statement [5].

This review is part of a broader systematic review focusing on the prevalence, clinical manifestations, diagnostic tests, and therapeutic strategies for DH. The protocol for the entire systematic review has been registered in PROSPERO (CRD42023444060).

The PICO (Population (P)—Patients with DH, regardless of age, gender, and severity of symptoms; Intervention (I)—Serological tests used to diagnose DH, including tests for IIF for IgA EMA and ELISA for IgA anti-tTG or IgA anti-eTG; Comparison (C)—The gold standard diagnostic methods for DH, including DIF of skin biopsies or absence of skin lesions; Outcome (O)—The primary outcome is the diagnostic accuracy of the serological tests, measured by sensitivity, specificity or both.

### Aim

This systematic review and meta-analysis aim to determine the diagnostic accuracy of serological tests for DH. Specifically, we will evaluate the quality of the available evidence, compare the aggregated sensitivities and specificities of different testing methods, and identify study, test, and the patient characteristics that influence the accuracy of these tests. To assess the impact of a GFD on test performance three separate meta-analyses were conducted. For the primary meta-analysis, we included patients with CeD who adhere to a GFD (Sample GFD). The secondary meta-analysis focused on patients with CeD at follow-up adhering to a GFD. The third meta-analysis compared DH patients on GFD at diagnosis with those with skin diseases or healthy individuals.

### Eligibility criteria

Studies were included in this systematic review if they met all the following eligibility criteria: (i) evaluation of the serological diagnostic method; (ii) aimed at the diagnosis of DH; (iii) the reference test was DIF or absence of skin lesions; (iv) only studies published in English. For the primary meta-analysis, we included only studies that involved patients at the time of diagnosis, not following a GFD. In the secondary meta-analysis, we included CeD patients at follow-up, who follow a GFD. In the third meta-analysis, we included only studies involving DH patients following a GFD at the time of diagnosis and patients with skin disease or healthy patients.

### Information source

PubMed, SCOPUS, EMBASE, and Web of Science were comprehensively searched from the inception. These databases were selected to ensure a broad and comprehensive coverage of relevant literature. The last search was conducted on July 14, 2023.

### Search

The search strategy utilised a combination of relevant descriptors, including 'Dermatitis herpetiformis', 'Duhring disease', and other related terms, combined with Boolean operators AND and OR to refine the search. The search was designed without combining accuracy terms to be more comprehensive. The complete search strategy is available in Table S1 (see Additional file 1).

### Study selection

The process of selecting the studies involved several stages. Initially, titles and abstracts of all retrieved articles were evaluated for eligibility using Covidence, a web-based collaboration software platform (Covidence systematic review software, Veritas Health Innovation, Melbourne, Australia. Available at www.covidence.org). Articles deemed potentially relevant were then read in full. Studies meeting the inclusion criteria were included in the systematic review, and, where applicable, in the meta-analysis. We excluded review articles, editorials, case reports, modeling or economic studies, and studies that only reported analytical sensitivity. Two authors (H.O. and G.B.) independently performed all steps of the literature selection process, discussing any discrepancies with a third author (F.Z.) to reach a consensus.

### Data collection process

Data extraction from selected reports was conducted using pilot forms to ensure consistency and accuracy. This process was performed independently and in duplicate by two authors. Any differences in the extracted data were resolved through discussion, and, if necessary, consultation with a third author.

### Data extraction

Data were extracted independently by two researchers using Covidence data extraction form. We categorised the tests by method, including IIF, ELISA, enzyme immunoassays (EIA), and immunoblotting (IB). In several studies, investigators evaluated the performance of more than one test method (e.g., ELISA and IIF) or more than one index test. For each index test performed in a study, we extracted the numbers needed to construct 2 × 2 contingency tables (true positives [TP], true negatives [TN], false positives [FP], false negatives [FN]). Each evaluation of a particular index test was considered its own study arm.

General study details included the authors, year of publication, country of origin, study design, and sample size were extracted. Information on the methods used, specifically categorizing the types of tests by their method, such as IIF, ELISA, EIA, and IB were collected. Characteristics of the study groups were recorded, including age (mean/median, range), the percentage of females, characteristics of the control group, GFD status, and whether the evaluation was concomitant with the first diagnosis. For each index test, we noted the instrument used, the type of immunoglobulin, the type of transglutaminases, and the cut-off for positivity. Diagnostic test results were compiled to include sensitivity, specificity, and accuracy.

### Risk of bias assessment

Two reviewers (H.O. and G.B.) independently evaluated the risk of bias in each study using the Diagnostic Precision Study Quality Assessment Tool (QUADAS-2) [6] recommended by the Cochrane Collaboration. QUADAS-2 is structured to assess four domains of potential sources of bias in primary diagnostic studies: patient selection, index test, reference standard, and flow and timing. Each domain is assessed in terms of risk of bias, and the first three domains are also evaluated regarding concerns about applicability. These applicability concerns help to judge the overall risk of bias. The QUADAS-2 tool is applied in four phases: summarising the review question, tailoring the tool and producing review-specific guidance, constructing a flow diagram for the primary study, and judging bias and applicability. This tool allows for a more transparent rating of bias and applicability of primary diagnostic accuracy studies.

### Synthesis of results

In this meta-analysis, we compared the gold standard diagnostic test with the three major serological tests for DH: IIF microscopy for qualitative and semi-quantitative detection of EMA, and IgA-based ELISA to detect tissue transglutaminase (tTG) and epidermal transglutaminase (eTG).

### Meta-analyses

We performed a meta-analysis of studies including patients with DH and CeD patients without cutaneus involvement at the time of diagnosis (sample not-GFD). Separately, we analysed the studies including all DH and CeD patients without cutaneus involvement at follow-up adhering to a GFD (sample GFD), as GFD is known to influence the accuracy of serological tests [7, 8]. A third meta-analysis was performed for studies including DH patients adhering to a GFD and patients with skin disease or healthy subjects (sample DH-GFD and SD or Healthy).

The index tests considered in the meta-analysis were chosen based on their prevalent use in clinical practice [3]. These tests comprised IIF for EMA detection and IgA-based ELISA for tTG or eTG detection.

The MetaBayesDTA web-based R Shiny application [9] was used for the meta-analysis. This tool, based on MetaDTA [9] and powered by Stan, implements Bayesian meta-analyses to measure the accuracy of diagnostic tests assuming an imperfect gold standard as a reference test. Given the aforementioned variability in DIF results, this Bayesian approach is particularly suitable, as it allows for a more nuanced analysis of the accuracy of serological tests in diagnosing DH [3].

We employed the "Latent Class Model" for this meta-analysis, which evaluates the accuracy of tests when a perfect gold standard does not exist. This Bayesian probabilistic model relates the index test, the reference test (one or more), and the latent disease state. It accounts for the imperfect reference test by estimating the true disease status of each participant using results from both the index and reference tests. Unlike traditional bivariate models, this model does not assume perfect accuracy of the reference test. Each test is assumed to measure the same latent disease, classifying each subject into either the diseased or non-diseased group, providing an estimate of the new diagnostic test's sensitivity and specificity [10, 11].

Sensitivity measures the test's ability to correctly identify individuals with the disease (true positives), while specificity measures its ability to correctly identify those without the disease (true negatives). To present these results effectively, pooled sensitivity and specificity estimates were calculated using the latent true disease status as the criterion standard. We calculated the Credible interval (CrI) for each result to characterise the posterior probability distribution in Bayesian statistics.

Initially, fixed values of sensitivity and specificity of the reference test were set between the studies analysed. A sample from the posterior distributions of the parameters of interest was obtained using a Monte Carlo Markov Chain (MCMC) algorithm via Stan. The Stan sampler options were set by default as follows: maximum treedepth of 10; target average proposal acceptance probability (adapt_delta) of 0.8; 2 sampling chains, with 1500 iterations each; and pooled estimates obtained by removing the first 500 iterations from each chain (warm-up iterations).

Furthermore, we computed pooled positive (LR +) and negative likelihood ratios (LR-), as well as diagnostic odds ratios (OR) with their 95% CrI, because diagnostic OR is a single indicator of test performance that combines sensitivity and specificity. Summary receiver operating curves (SROC) were fashioned, with the horizontal axis of the ROC space representing the false positive rate (1-specificity) and the vertical axis representing the sensitivity of primary studies. The placement of points on the plot provides insight into the trade-off between sensitivity and specificity: points closer to the top-left corner (high sensitivity and low false positive rate) indicate better diagnostic accuracy. Conversely, points further away from this corner suggest reduced performance. The size of the circles represents the weight of individual studies, typically determined by their sample size, with larger circles corresponding to studies with greater influence on the meta-analysis results. The dashed circles or ellipses represent the 95% prediction interval (PI), which visualizes the range of variability expected in future studies. A narrower prediction interval indicates more consistent performance across studies, while a broader interval reflects higher heterogeneity.

We synthesised the results descriptively for studies that did not meet the criteria for inclusion in the meta-analyses. This synthesis included a qualitative comparison of the study characteristics, populations, and outcomes. We systematically reviewed the sensitivity and specificity reported in these studies, providing a narrative summary of their findings. Where possible, we compared these results with those of the meta-analyses to highlight any significant deviations or trends.

## Results

### Study selection

**Fig. 1:**
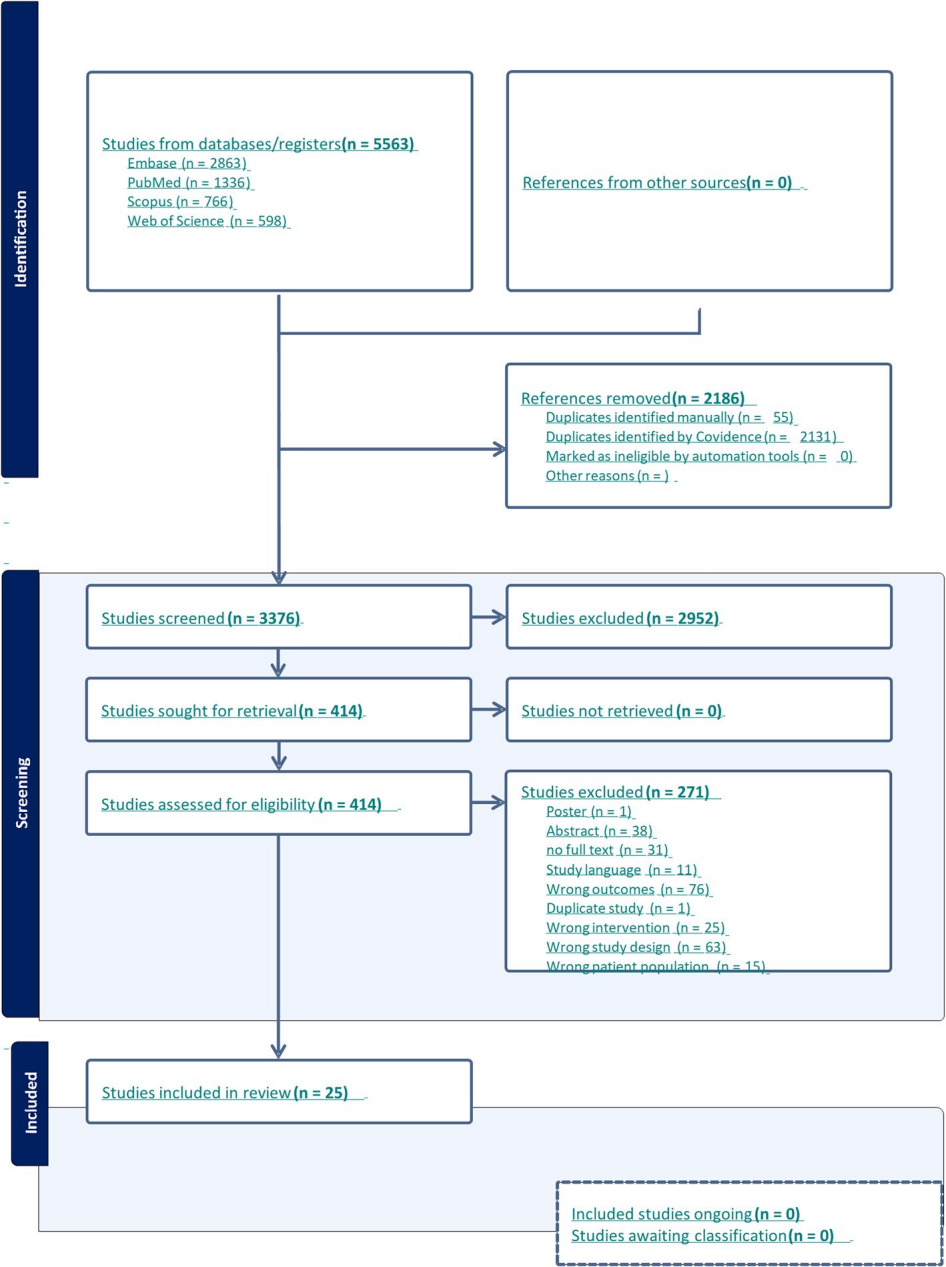
PRISMA Flowchart

A total of 5563 studies were initially screened for the overall review. After assessing the titles and abstracts for eligibility, 2952 studies were excluded based on predefined criteria, such as review articles, editorials, case reports, and modeling or economic studies. The full texts of the remaining 414 studies were then reviewed for all research questions. For this specifc review we include 25 studies and 9 of these were considered in the meta-analysis (Fig. 1).

### Study characteristics

The studies were conducted across various countries, including Italy (32%), the USA (28%), Finland (16%), Poland (16%), Germany (8%), Austria (4%), Bulgaria (4%), Argentina (4%) and Hungary (8%), with Colombia (4%) having the lowest frequency. The study designs included a variety of approaches: retrospective (52%); prospective (28%); cross-sectional (12%); and case series (8%). Participants included patients with suspected or confirmed DH. Some studies specifically focused on those following a GFD, while others examined patients at different diagnostic stages. Sample size varied significantly, with studies that included as few as 11 participants [12] to as many as 411 participants [13] (Table 1).

**Table 1:** Characteristics of the included studies

Author	Country	Study design	GFD y/n	Diagnosis at baseline	Sample size	Dermatitis group	N Female DH	Age DH median (range)	Control group	N control group	N Female control	Age control median (range)
Antiga et al*.*, 2021 [14]	Italy	Prospective	No	Yes	45	29	17	NA	CeD patients	16	12	NA
Biagi et al*.*, 2003 [30]	Italy	Case series	Mixed	Yes	27	11	2	33,4 †	HP	16	8	31,1 †
Bonciolini et al*.*, 2019 [24]	Italy	Prospective	Mixed	Na	18	0	NA	NA	18 SD patients	18	16	NA
Borroni et al*.*, 2013 [19]	Italy	Retrospective	No	Na	308	44	19	38 (16–76)	99 CeD patients, 70 SD patients, 95 GI controls	264	125	13–92
Bresler and Granter, 2015 [31]	USA	Retrospective	Mixed	Yes	77	16	NA	NA	Clinical suspicion of DH	61	NA	NA
Cannistraci et al*.*, 2007 [12]	Italy	Prospective	Mixed	Yes	11	2	0	42,5	CeD patients	9	8	NA
Caproni et al*.*, 2021 [15]	Italy	Prospective	No	Na	32	19	12	45,84 † (9–77)	CeD patients	13	6	36,85 † (17–70)
Dahlbom et al*.*, 2010 [23]	Finland, Sweden	Retrospective	Mixed	Na	265	134	NA	9,5 (2,4–68,8)	CeD patients and HP	131	NA	10,8 (0,8–58,2)
Gornowicz-Porowska et al*.*, 2012 [32]	Poland	Retrospective	No	Na	80	31	12	NA	25 SD patients, 24 HP	49	NA	NA
Gornowicz-Porowska et al*.*, 2021 [28]	Poland	Retrospective	No	Na	55	31	17	40 † (9–80)	HP	24	NA	NA
Hadjivassiliou et al*.*, 2020 [25]	Finland	Retrospective	Mixed	Yes	69	33	NA	NA	36 SD patients	36	NA	NA
Heil et al*.*, 2005 [18]	Austria	Retrospective	Mixed	No	103	29	12	41,6 † (17–87)	54 HP, 20 CeD patients	74	49	NA
Hull et al*.*, 2008 [22]	USA	Cross-sectional	Mixed	Yes	153	44	NA	NA(19–80)	19 CeD patients, 37 SD patients, 53 HP	109	NA	NA
Jaskowski et al*.*, 2010 [29]	USA	Retrospective	Mixed	Na	129	80	NA	NA	HP	49	NA	NA
Kasperkiewicz et al*.*, 2012 [27]	Germany	Prospective	Mixed	Na	97	45	20	54 (10–86)	52 SD patients	52	NA	NA
Kumar et al*.*, 2001 [17]	Poland, USA	Retrospective	Mixed	Na	369	44	NA	NA	58 CeD patients, 161 SD patients, 106 HP	325	NA	NA
Lytton et al*.*, 2013 [20]	Italy, Poland, USA	Retrospective	Mixed	Na	200	93	30	NA (21–80)	SD patients	107	73	47 † (18–101)
Marietta et al*.*, 2008 [26]	USA, Colombia	Case series	Yes	No	24	9	4	68 (35–75)	CeD patients	15	10	52 (13–71)
McCord and Hall III, 2009 [33]	USA	Cross-sectional	Yes	Na	13	8	0	NA	HP	5	0	NA
Nätynki et al*.*, 2020 [40]	Finland	Retrospective	Mixed	Na	94	46	16	48,6 †	HP	48	31	49,4 †
Sárdy et al*.*, 2000 [16]	Hungary	Retrospective	Mixed	Na	380	47	23	30,2 † (8,8–71)	120 CeD patients, 96 non-CeD patients, 117 other diseases	333	181	(0,4–78)
Sárdy et al*.*, 2002 [34]	Hungary, Germany	Retrospective	Mixed	Na	215	59	28	30,8 † (6,2–73,5)	79 GI diseases, 47 other diseases, 30 HP	156	81	10,2 (0,5–55,5)
Sugai et al*.*, 2006 [13]	Argentina	Prospective	No	Na	411	18	9	38 † (14–72)	No-CeD patients	391	260	46 † (16–87)
Velikova et al*.*, 2019 [35]	Bulgaria	Prospective	No	Na	46	26	NA	53 † (18–72)	HP	20	NA	31 † (24–42)
Ziberna et al*.*, 2021 [21]	Italy, Finland	Cross-sectional	Unclear	Na	214	46	NA	NA	HP	168	NA	NA

*Abbreviations*: *CeD* Celiac disease, *HP* Healthy patients, *GI* Gastrointestinal, *Na* Not available, *SD* Skin disease, †mean of age

The studies included in the meta-analyses were reported in Table 2, they were 2 prospective [14, 15]; 6 retrospective [16–21] and 1 cross-sectional [22]. Among the DH groups, female representation ranged from 26 to 63%, and mean ages varied, typically falling between 30.2 and 51.0 years. The studies included had one group of patients with a diagnosis of DH through DIF analysis and at least one group of patients without DH diagnosis through DIF analysis or absence of skin lesions.The exception is Antiga et al. that has only one group of CeD untreated without skin lesions who were analyzed with a serological test for CeD [14].

**Table 2:** Diagnostic test characteristics in studies included in the meta-analysis

Author	Population case	Population control	Sample Size	N DH patients	N no-DH patients	Reference	Index	Immunoglobulin	Protein	Sensitivity (%)	Specificity (%)
Antiga [14]	DH at diagnosis	CeD at diagnosis	45	0	45	DIF or absence of skin lesions	ELISA	IgA	tTG	NA	6,67
									eTG	NA	77,78
Borroni [19]	DH at diagnosis	CeD at diagnosis	143	44	99	DIF or absence of skin lesions	IIF	IgA	EMA	NA	0,00
Caproni [15]	DH at diagnosis	CeD at diagnosis	32	19	13	DIF or absence of skin lesions	ELISA	IgA	tTG	68,42	0,00
									eTG	57,89	76,92
Heil [18]	DH at diagnosis	CeD at diagnosis	49	29	20	DIF or absence of skin lesions	ELISA	IgA	tTG	96,55	5,00
									eTG	44,83	85,00
Hull [22]	DH at diagnosis	CeD at diagnosis	63	44	19	DIF or absence of skin lesions	ELISA	IgA	tTG	45,45	21,05
									eTG	52,27	47,37
Kumar [17]	DH at follow up	SD or HS	278	11	267	DIF or absence of skin lesions	ELISA	IgA	tTG	27,27	97,00
							IIF	IgA	EMA	0,00	100,00
	DH at follow up	CeD at follow up	35	11	24	DIF or absence of skin lesions	IIF	IgA	EMA	0,00	87,50
							ELISA	IgA	tTG	27,27	95,83
	DH at diagnosis	CeD at diagnosis	67	33	34	DIF or absence of skin lesions	IIF	IgA	EMA	100,00	0,00
							ELISA	IgA	tTG	100,00	8,82
Lytton [20]	DH at follow up	SD or HS	147	40	107	DIF or absence of skin lesions	ELISA	IgA	tTG	40,00	100,00
									eTG	42,50	87,36
Sardy [16]	DH at follow up	SD or HS	229	16	213	DIF or absence of skin lesions	ELISA	IgA	tTG	43,75	98,59
							IIF	IgA	EMA	43,75	100,00
	DH at diagnosis	CeD at diagnosis	79	31	48	DIF or absence of skin lesions	IIF	IgA	EMA	93,55	18,75
							ELISA	IgA	tTG	90,32	20,83
	DH at follow up	CeD at follow up	88	16	72	DIF or absence of skin lesions	IIF	IgA	EMA	43,75	52,78
							ELISA	IgA	tTG	43,75	62,50
Ziberna [21]	DH at follow up	SD or HS	214	46	168	DIF or absence of skin lesions	ELISA	IgA	eTG	82,61	90,48

*Abbreviations: CeD* Celiac disease, *HP* Healthy patients, *GFD* Gluten Free Diet, *DIF* Direct immunofluorescence, *IIF* Indirect immunofluorescence, *ELISA* Enzyme-Linked Immunosorbent Assay, *EMA* Endomysial, *IgA* Immunoglobulin A, *tTG* tissutal transglutaminase, *eTG* epidermal transglutaminase, *SD* Skin disease, *HS* Healthy subjects, *NA* Not available

The studies included in the meta-analysis with at least one patient under 18 years of age are 3 [15, 16, 19].

### Diagnostic accuracy of serological tests for dermatitis herpetiformis

Table 2 reports the information on the diagnostic precision of serological tests for the study included in the meta-analyses. The index tests most commonly evaluated were ELISA for IgA tTG appearing in 7 (100%) [14–18, 22, 23], follow by ELISA for IgA eTG that appear in 6 (67%) [14, 15, 18, 20–22]. IIF for IgA EMA was used in 3 (33%) [16, 17, 19] studies.

Table S2 (see Additional file 1) reports other serological diagnostic tests not included in the meta-analysis due to insuddicient data. These tests, while potentially informative, are less commonly used in routine diagnostic workflows compared to other serological methods. They were: ELISA for antibody DGP, AGA, TG6, nGli, GAF3X, Glia or AAA based on IgA, IgG or in combination IgA and IgG; IB for tTG, AGA or npG of type IgA or IgG; EIA for tTG, DGP based on IgA, IgG or in combination IgA and IgG.

IIF for IgA EMA tests showed a sensitivity range from 90.91% [19] to 100% [17] for ‘sample at diagnosis not-GFD’ and a specificity range from 0.00% [17, 19] to 18.75% [16]; instead, for ‘sample at follow-up GFD’ sensitivity ranged from 0.00% [17] to 43.75% [16] and specificity ranged from 52.78% [16] to 87.50% [17]. Finally, for ‘sample DH at follow up-GFD and SD or Healthy’ sensitivity ranged from 0.00% [17] to 43.75% [16], and specificity was 100% for both studies included [16, 17].

ELISA for IgA tTG tests showed a sensitivity range from 45.45% [22] to 100% [17] for ‘sample at diagnosis no-GFD’, and a specificity range from 0% [15] to 21.05% [22]; instead, for ‘Sample GFD’ sensitivity ranged from 27.27% [17] to 43.75% [16] and specificity ranged from 62.50% [16] to 95.83% [17]. Finally, for ‘sample DH at follow up-GFD and SD or healthy’ sensitivity ranged from 27.27% [17] to 43.75% [16], and specificity ranged from 97.00% [17] to 100.00% [20].

ELISA for IgA eTG, for ample at diagnosis no-GFD’ sensitivity ranged from 34.48% [14] to 57.89% [15] and specificity ranged from 47.37% [22] to 100% [14]. Instead, for ‘Sample at follow-up GFD’, no studies evaluated the accuracy of ELISA for IgA eTG in celiac patients adhering to GFD. Finally, for ‘Sample DH at follow up-GFD and SD or Healthy’ sensitivity ranged from 42.50% [20] to 82.61% [21] and specificity ranged from 87.36% [20] to 90.48% [21].

### Risk of bias and applicability

The evaluation of the risk of bias and concerns regarding applicability for each study is shown in Fig. S1 and S2.

The risk of bias assessment in the studies included in the meta-analysis highlighted significant issues in patient selection (D1) and index test (D2) domains (Fig. S1, see Additional file 1). High risk was noted in 2 studies [16, 19], indicating concerns about the representativeness of study populations and the application or interpretation of index tests; instead, Caproni et al. [15], Heil et al. [18], Hull et al. [22], Kumar et al. [17] and Lytton et al. [20] had high risk in patient selection and some concerns about index test. The reference standard (D3) and flow & timing (D4) domains generally showed lower risk, but Sardy et al. [16] and Lytton et al. [20] had high risk in flow & timing, suggesting issues with test timing and participant flow.

The risk of bias assessment in the studies not included in the meta-analysis also highlighted issues in patient selection (D1) and index test (D2) domains (Fig. S2, see Additional file 1). High risk was noted in all the studies examined, except Bonciolini et al. [24], indicating concerns about the representativeness of study populations and the application or interpretation of index tests. The reference standard (D3) and flow & timing (D4) domains generally showed a lower risk. However, some studies, such as Hadjivassiliou et al. [25] and Marietta et al. [26], exhibited high risk in the flow & timing domain, suggesting test timing and participant flow issues. Finally, 5 studies an overall showed high risk [23, 25–28], and 3 studies showed an overall low risk [12, 24, 29].

### Meta-analysis on sample at diagnosis not-GFD – comparison of three serological tests

**Fig. 2:**
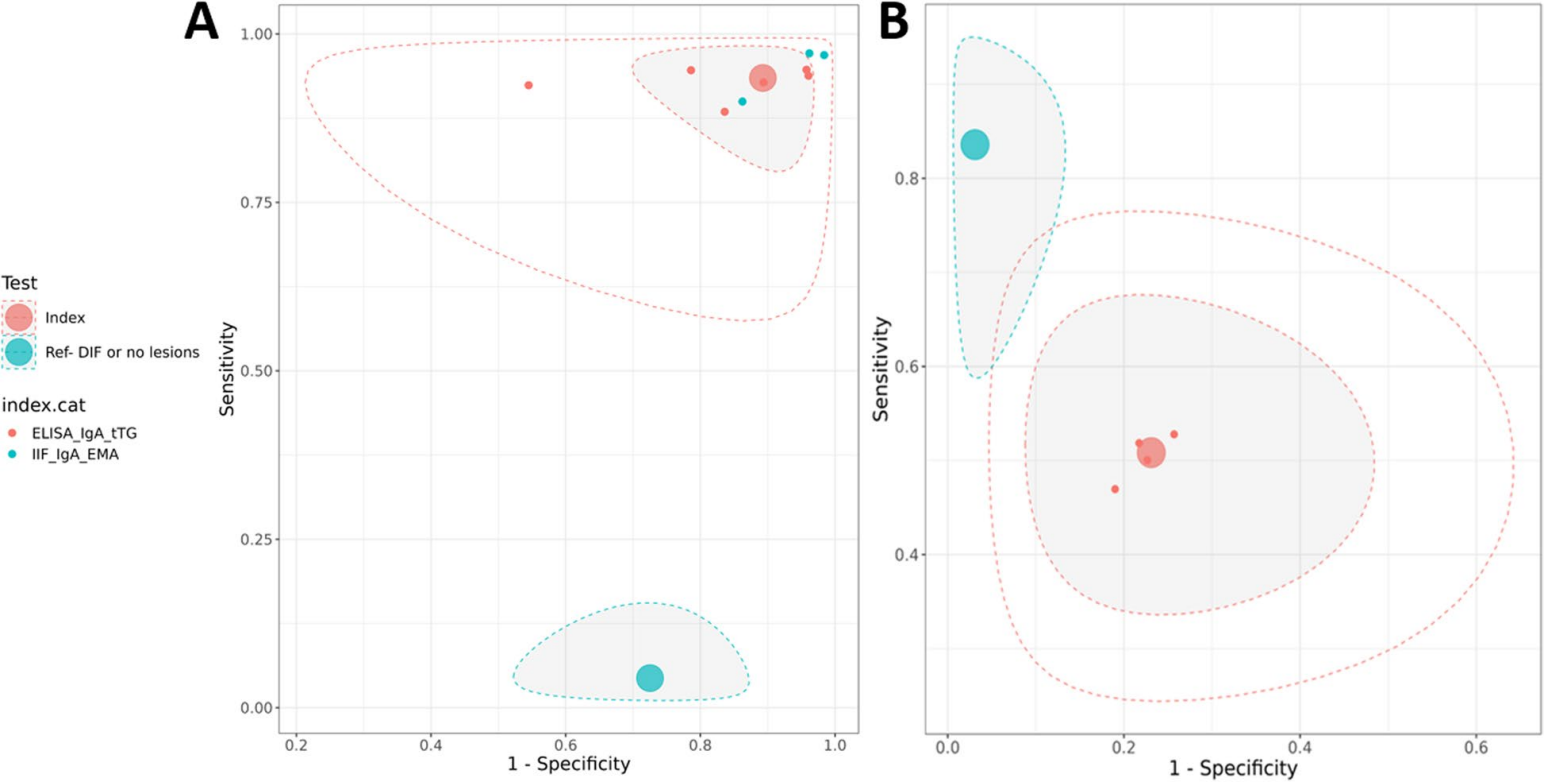
Plots of diagnostic accuracy in DH and CeD patients at diagnosis. Summary Receiver Operating Characteristic (SROC) plot for latent class model analysis of IIF for IgA EMA and ELISA for IgA tTG (**A**), and ELISA forIgA eTG (**B**) compared to DIF or absence of skin lesions in DH and CeD patients at diagnosis

The analysis includes only CeD patients who had not adhered to GFD, or who had been on GFD for less than one month or who were undergoing a gluten challenge (n = 7). Figure 2 presents the SROC plots from the latent class model analysis for the diagnostic performance aggregated of IIF for IgA EMA with ELISA for IgA tTG (A) and ELISA for IgA eTG (B) compared to DIF in diagnosing dermatitis herpetiformis.

The aggregated diagnostic accuracy of IIF for IgA EMA with ELISA for IgA tTG, and ELISA for IgA eTG tests was evaluated and compared to the DIF reference standard or absence of skin lesions. For IIF for IgA EMA with ELISA for IgA tTG, the SE was 0.94 (95% CrI: 0.82, 0.98), and SP was 0.11 (95% CrI: 0.03, 0.28) (Table 3). Still, the value of SP is very low, as indicated by its proximity to the top right corner of the ROC space (Fig. 2A). This suggests that these tests are valid tests for diagnosing patients with high suspicion of having DH. The ELISA for IgA eTG exhibited the most balanced results between sensitivity and specificity: with higher specificity (0.77, 95% CrI: 0.50, 0.92) than the others but lower sensitivity (0.51, 95% CrI: 0.32, 0.69) (Table 3).

**Table 3:** Results of diagnostic accuracy in DH and CeD patients at diagnosis

Test	Parameter	IIF for IgA EMA and ELISA for IgA tTG			ELISA for IgA eTG		
		**Median**	**SD**	**95% Credible Interval**	**Median**	**SD**	**95% Credible Interval**
**Index**	Sensitivity	0.94	0.05	(0.82, 0.98)	0.51	0.09	(0.32, 0.69)
	Specificity	0.11	0.07	(0.03, 0.28)	0.77	0.11	(0.50, 0.92)
	False Positive Rate	0.89	0.07	(0.72, 0.97)	0.23	0.11	(0.08, 0.50)
	Diagnostic Odds Ratio	1.74	3.94	(0.24, 12.48)	3.40	3.64	(0.82, 14.52)
	Likelihood Ratio +	1.05	0.12	(0.88, 1.30)	2.18	1.48	(0.90, 6.67)
	Likelihood Ratio -	0.61	1.04	(0.10, 3.80)	0.65	0.21	(0.39, 1.07)
	Between-study Correlation	−0.19	0.40	(−0.83, 0.63)	−0.02	0.45	(−0.83, 0.80)
	Cutpoint parameter	2.64	1.45	(1.73, 6.03)	−0,54	0.92	(−2.94, 0.46)
	Accuracy parameter	1.76	3.69	(−1.40, 10.96)	1.15	1.80	(−0.80, 5.98)
	Shape parameter	0.49	0.82	(−0.65, 2.68)	0.44	1.66	(−2.99, 3.77)
	SD of cutpoint parameter	0.68	0.58	(0.06, 2.32)	0.06	0.21	(0.00, 0.75)
	SD of accuracy parameter	2.73	2.33	(0.22, 9.30)	0.25	0.85	(0.00, 2.99)
	Between-study SD for logit(Sensitivity)	0.90	0.52	(0.10, 2.15)	0.32	0.39	(0.02, 1.45)
	Between-study SD for logit(Specificity)	1.45	0.45	(0.78, 2.54)	0.52	0.53	(0.02, 1.99)
**Reference**	Sensitivity	0.04	0.03	(0.01, 0.13)	0.84	0.08	(0.68, 0.97)
	Specificity	0.28	0.08	(0.10, 0.40)	0.97	0.03	(0.90, 0.99)
	False Positive Rate	0.73	0.08	(0.60, 0.90)	0.03	0.03	(0.01, 0.10)
	Diagnostic Odds Ratio	0.02	0.02	(0.00, 0.07)	172.05	826.68	(30.46, 1792.53)
	Likelihood Ratio +	0.06	0.04	(0.01, 0.18)	26.48	51.20	(8.03, 142.00)
	Likelihood Ratio -	3.45	5.34	(2.37, 10.53)	0.17	0.08	(0.04, 0.34)

For IIF for IgA EMA and ELISA for IgA tTG, the diagnostic OR was 1.74 (95% CRI: 0.24, 12.48), suggesting good diagnostic performance but with a large CrI. Instead, ELISA for IgA eTG showed a diagnostic OR of 3.40 (95% CRI: 0.82, 14.52) (Table 3).

The FPR is the probability that a positive result will be given when the true value is negative. The FPR for IIF for IgA EMA and ELISA for IgA tTG was 0.89 (95% CrI: 0.72, 0.97), and for ELISA for IgA eTG was 0.23 (95% CrI: 0.08, 0.50) (Table 3).

The LRs provide additional insights into the test’s diagnostic utility. The LR + for IIF for IgA EMA and ELISA for IgA tTG was 1.05 (95% CrI: 0.88, 1.30), and LR- was 0.61 (95% CrI: 0.10, 3.80). Instead, the LR + for ELISA for IgA eTG was 2.18 (95% CrI: 0.90, 6.67), and the LR- was 0.65 (95% CrI: 0.39, 1.07) (Table 3).

### Meta-analysis at follow-up on sample GFD – comparison of three serological tests

**Fig. 3:**
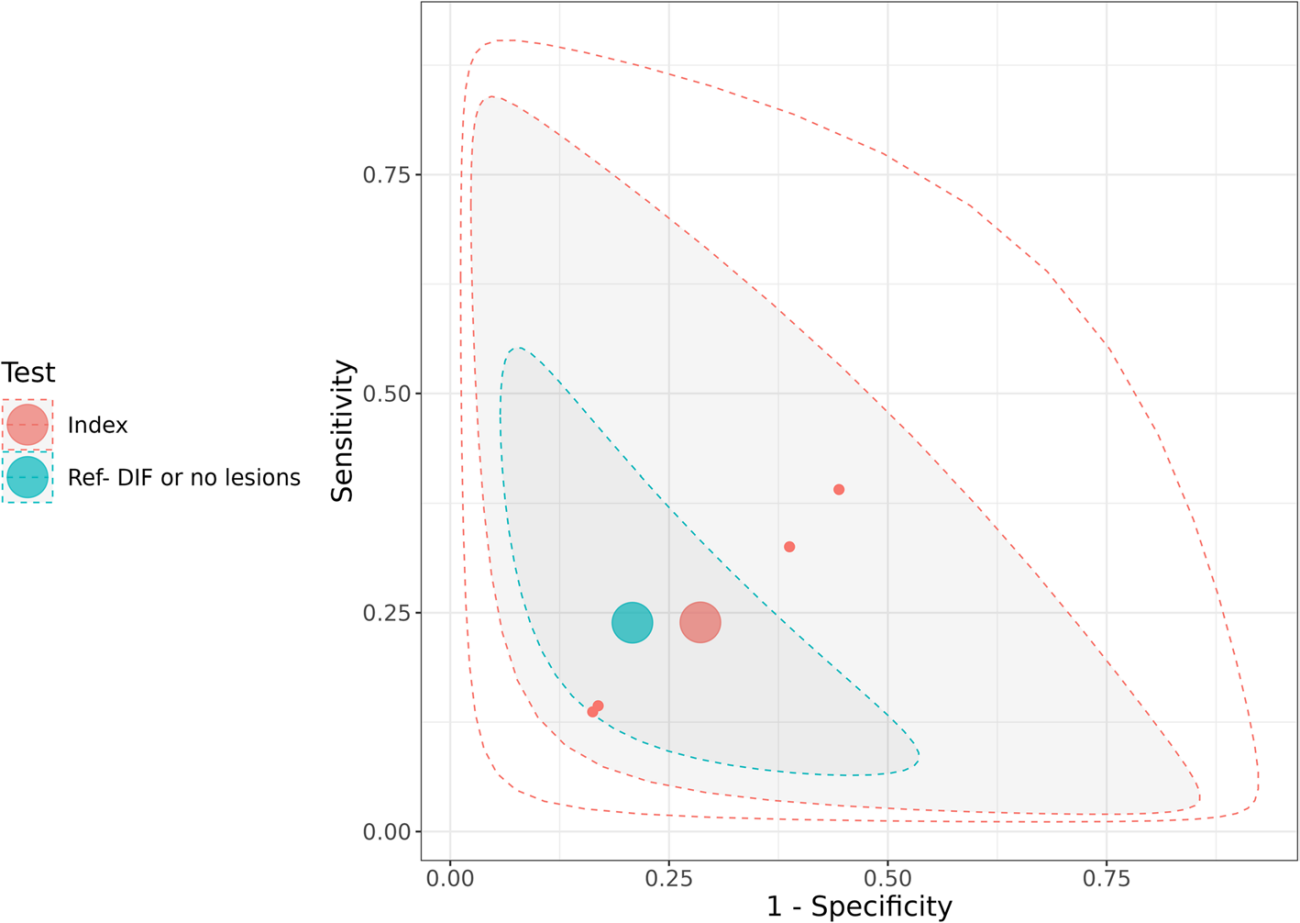
Plot of diagnostic accuracy in DH and CeD patients at follow up in GFD. Summary Receiver Operating Characteristic (SROC) plot for latent class model analysis of IIF for IgA EMA with ELISA for IgA tTG compared to DIF or absence of skin lesion in DH and CeD patients at follow up

The analysis includes DH patients during follow-up who were on a GFD in comparison with CeD patients without cutaneus lesions on GFD (n = 2). Figure 3 presents the SROC plots from the latent class model analysis for the aggregated diagnostic performance of IIF for IgA EMA with ELISA for IgA tTG.

The aggregated diagnostic accuracy of IIF for IgA EMA with ELISA for IgA tTG was evaluated and compared to the DIF reference standard or the absence of skin lesions. For IIF for IgA EMA with ELISA for IgA tTG, the SE was 0.24 (95% CrI: 0.03, 0.79), and SP was 0.71 (95% CrI: 0.21, 0.97) (Table 4).

**Table 4:** Results of diagnostic accuracy in DH and CeD patients at follow up in GFD

Test	Parameter	IIF for IgA EMA and ELISA for IgA tTG		
		**Median**	**SD**	**95% Credible Interval**
**Index**	Sensitivity	0.24	0.22	(0.03, 0.79)
	Specificity	0.71	0.22	(0.21, 0.97)
	False Positive Rate	0.29	0.22	(0.04, 0.79)
	Diagnostic Odds Ratio	0.76	32.81	(0.020, 49.44)
	Likelihood Ratio +	0.81	5.17	(0.06, 15.55)
	Likelihood Ratio -	1.07	1.60	(0.25, 4.34)
	Between-study Correlation	−0,04	0.44	(−0.82, 0.79)
	Cutpoint parameter	−1,09	2.60	(−5.68, 0.57)
	Accuracy parameter	−0,28	5.98	(−7.82, 7.49)
	Shape parameter	0.01	1.62	(−3.22, 3.29)
	SD of cutpoint parameter	0.18	0.42	(0.00, 1.54)
	SD of accuracy parameter	0.72	1.66	(0.01, 6.15)
	Between-study SD for logit(Sensitivity)	0.70	0.58	(0.03, 2.16)
	Between-study SD for logit(Specificity)	0.72	0.60	(0.03, 2.21)
**Reference**	Sensitivity	0.24	0.11	(0.06, 0.48)
	Specificity	0.79	0.11	(0.54, 0.94)
	False Positive Rate	0.21	0.11	(0.06, 0.46)
	Diagnostic Odds Ratio	1.24	3.94	(0.10, 10.47)
	Likelihood Ratio +	1.18	2.09	(0.16, 6.34)
	Likelihood Ratio -	0.96	0.33	(0.59, 1.66)

*Abbreviations: IIF* Indirect immunofluorescence, *ELISA* Enzyme-Linked Immunosorbent Assay, *EMA* Endomysial, *IgA* Immunoglobulin A, *tTG* tissutal transglutaminase, *SD* standard deviation

For IIF for IgA EMA and ELISA for IgA tTG, the diagnostic OR was 0.76 (95% CRI: 0.02, 49.44), suggesting unsatisfactory diagnostic performance.The FPR for IIF for IgA EMA and ELISA for IgA tTG was 0.29 (95% CrI: 0.04, 0.79 (Table 3).The LR + for IIF for IgA EMA and ELISA for IgA tTG was 0.81 (95% CrI: 0.06, 15.55), and LR- was 1.07 (95% CrI: 0.25, 4.34) (Table 3).

### Meta-analysis on DH sample at follow-up-GFD and skin disease or healthy – comparison of three serological tests

**Fig. 4:**
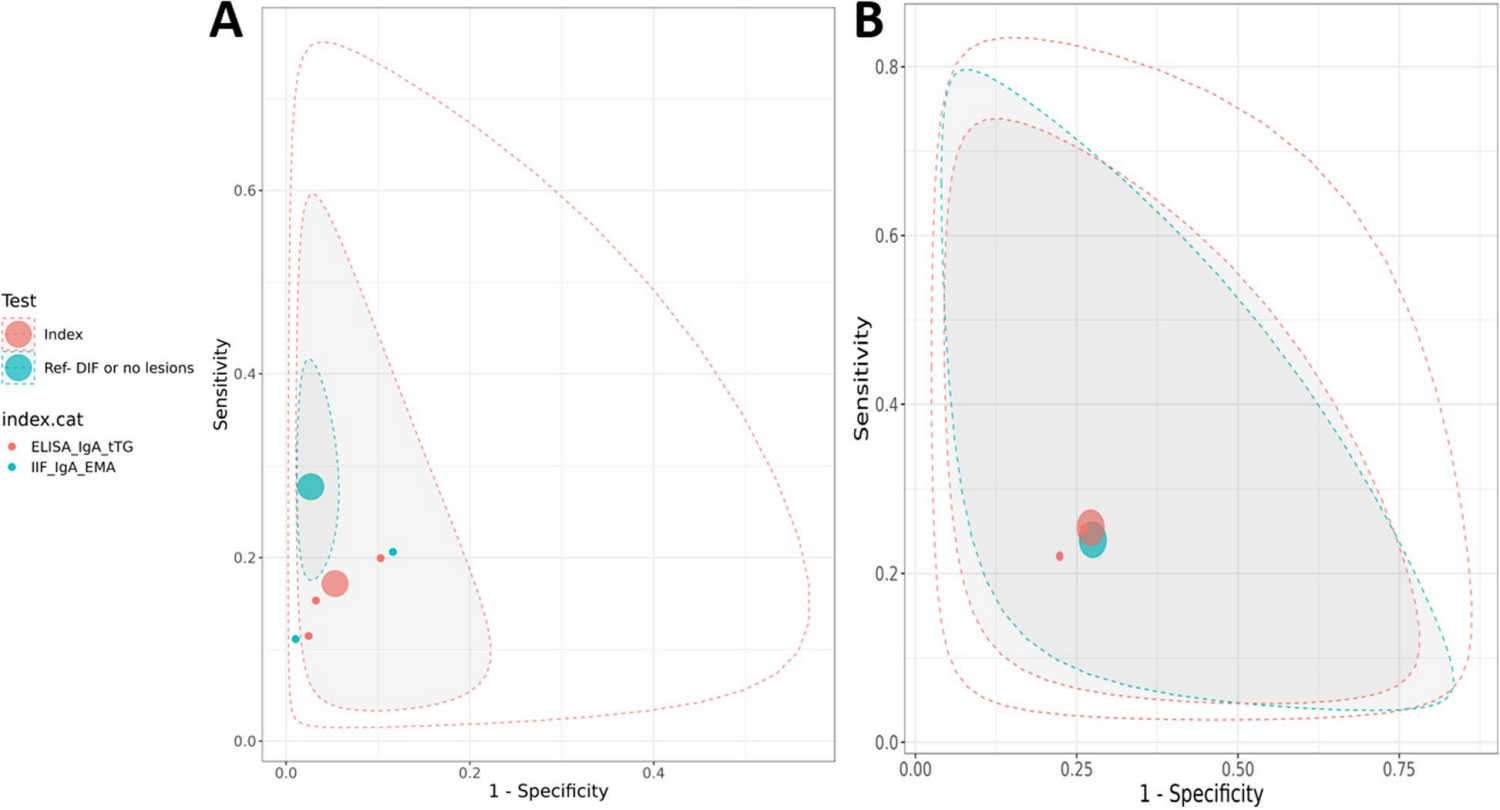
Plots of diagnostic accuracy in DH patients at follow up in comparison to no DH. Summary Receiver Operating Characteristic (SROC) plot for latent class model analysis of IIF for IgA EMA and ELISA for IgA tTG (**A**), and ELISA forIgA eTG (**B**) compared to DIF or absence of skin lesions in DH patients at follow up in comparison to patients with skin diseases (no DH) and healthy patients

The analysis includes DH patients on a GFD in comparison with patients with other skin diseases or healthy subjects. Figure 4 presents the SROC plots from the latent class model analysis for the aggregated diagnostic performance of IIF for IgA EMA with ELISA for IgA tTG (A), and ELISA for IgA eTG (B) compared to DIF in diagnosing dermatitis herpetiformis.

The aggregated diagnostic accuracy of IIF for IgA EMA with ELISA for IgA tTG, and ELISA for IgA eTG tests was evaluated and compared to the DIF reference standard or the absence of skin lesions. For IIF for IgA EMA with ELISA for IgA tTG, the SE was 0.17 (95% CrI: 0.06, 0.71), and SP was 0.95 (95% CrI: 0.80, 0.99) (Table 5). However, the value of SP is very high, as indicated by its proximity to the bottom left corner of the ROC space (Fig. 4A). This suggests that these tests are valid to excludr the diagnosis of DH in patients. The ELISA for IgA eTG exhibited more balanced results between sensitivity and specificity: with a higher sensitivity (0.25, 95% CrI: 0.05, 0.80) than the others but lower specificity (0.73, 95% CrI: 0.19, 0.94) (Table 5).

**Table 5:** Results of diagnostic accuracy in DH patients at follow up in comparison to no DH

Test	Parameter	IIF for IgA EMA and ELISA for IgA tTG			ELISA for IgA eTG		
		**Median**	**SD**	**95% Credible Interval**	**Median**	**SD**	**95% Credible Interval**
**Index**	Sensitivity	0.07	0.13	(0.01, 0.49)	0.25	0.19	(0.05, 0.80)
	Specificity	0.93	0.14	(0.49, 0.99)	0.73	0.20	(0.19, 0.94)
	False Positive Rate	0.07	0.14	(0.01, 0.51)	0.27	0.20	(0.06, 0.81)
	Diagnostic Odds Ratio	1.01	23.70	(0.01, 67.84)	0.93	11.53	(0.03, 31.85)
	Likelihood Ratio +	1.01	14.29	(0.02, 40.94)	0.95	2.78	(0.11, 9.15)
	Likelihood Ratio -	1.00	0.40	(0.52, 2.01)	1.02	1.86	(0.25, 4.43)
	Between-study Correlation	−0,11	0.46	(−0.87, 0.79)	0.01	0.43	(−0.80, 0.81)
	Cutpoint parameter	−2,88	3.21	(−7.64, −0.92)	−1,22	2.61	(−4.29, 0.71)
	Accuracy parameter	0.07	7.54	(−10.37, 9.69)	−0,25	0.58	(−6.92, 6.11)
	Shape parameter	−0,02	1.63	(−3.35, 3.04)	0.08	1.56	(−2.88, 3.24)
	SD of cutpoint parameter	0.21	0.47	(0.01, 1.64)	0.13	3.39	(0.00, 1.27)
	SD of accuracy parameter	0.83	1.87	(0.02, 6.55)	0.51	1.36	(0.01, 5.09)
	Between-study SD for logit(Sensitivity)	0.76	0.63	(0.04, 2.43)	0.59	0.56	(0.03, 2.08)
	Between-study SD for logit(Specificity)	0.75	0.63	(0.04, 2.34)	0.64	0.54	(0.04, 1.96)
**Reference**	Sensitivity	0.12	0.25	(0.02, 0.94)	0.24	0.21	(0.04, 0.86)
	Specificity	0.89	0.27	(0.05, 0.98)	0.73	0.23	(0.10, 0.94)
	False Positive Rate	0.12	0.27	(0.02, 0.96)	0.28	0.23	(0.06, 0.90)
	Diagnostic Odds Ratio	2.03	238.23	(0.02, 511.09)	0.75	23.05	(0.01, 45.70)
	Likelihood Ratio +	1.80	12.60	(0.03, 32.16)	0.80	3.47	(0.07, 9.80)
	Likelihood Ratio -	1.01	10.76	(0.07, 21.24)	1.08	2.84	(0.17, 8.80)

*Abbreviations: IIF* Indirect immunofluorescence, *ELISA* Enzyme-Linked Immunosorbent Assay, *EMA* Endomysial, *IgA* Immunoglobulin A, *tTG* tissutal transglutaminase, *eTG* epidermal transglutaminase, *SD* standard deviation

For IIF for IgA EMA and ELISA for IgA tTG, the diagnostic OR was 3.66 (95% CRI: 0.46, 149.62), suggesting good diagnostic performance but with a large CrI. Instead, ELISA for IgA eTG showed a diagnostic OR of 0.93 (95% CRI: 0.03, 31.85) (Table 5).

The FPR is the probability that a positive result will be given when the true value is negative. The FPR for the couple IIF for IgA EMA and ELISA for IgA tTG was 0.88 (95% CrI: 0.67, 0.97), and for ELISA for IgA eTG was 0.44 (95% CrI: 0.03, 0.88) (Table 3).

The LRs provide additional insights into the diagnostic utility. The LR + for IIF for IgA EMA and ELISA for IgA tTG was 0.05 (95% CrI: 0.01, 0.20), and LR- was 0.88 (95% CrI: 0.30, 1.11). Instead, the LR + for ELISA for IgA eTG was 0.95 (95% CrI: 0.11, 9.15), and the LR- was 1.02 (95% CrI: 0.25, 4.43) (Table 5).

### Results for studies not included in the meta-analysis

The 16 studies not included in the meta-analysis had observed the results of serological diagnostic tests in different populations, or had other diagnostic tests than IIF for IgA EMA, ELISA for IgA tTG and ELISA for IgA eTG, or they had different aims than accuracy of serological tests (Table S2). The only study to compare pediatric patients with and without DH is Dahlbom et al. [23], which examined the accuracy of the ELISA IgA for tTG in these two groups of patients. Their analysis showed that 95% of pediatric patients with DH were positive on ELISA IgA for tTG, whereas 100% of pediatric patients with CeD and absence of skin lesions were positive on ELISA IgA for tTG [23].

Biagi et al. [30] studied the distribution of tTG in the skin of DH patients and HP to find a correlation between DH diagnosis and tTG expression in the skin. Bonciolini et al. [24] investigated whether IgA deposits could be found in the skin of CeD patients who do not have inflammatory DH. Bresler and Granter [31] examined the utility of DIF testing for the characteristic IgA deposits of DH in patients stratified into high and low clinical suspicion subgroups, and their results argued that it may be reasonable to first perform a duodenal biopsy for routine histologic evaluation before requesting DIF analysis. Cannistracci et al. [12] evaluated the presence of IgA and eTG deposits in healthy skin of CeD patients without cutaneous manifestations, so they demonstrated the presence of IgA and eTG deposits on healthy skin of celiac patients. Dahlbom et al. [23] analysed whether the quantification of autoantibodies against tissue transglutaminase could be used to predict mucosal destruction and disease severity in patients with gluten sensitivity, hence they found that high levels of IgA-tTG and IgG-tTG antibodies were associated with the grade of mucosal villous atrophy and a more severe clinical presentation, as in DH patients. Gornowicz-Porowska et al. [32] found statistically significant correlations between levels of anti-tTG/anti-npG IgA in DH on unclear diet patients and patients with IgA/neutrophilil-mediated non-DH dermatoses. A more recent study by Gornowicz-Porowska et al. [28] compared the utility of ELISA and IB with tTG in serological diagnoses of DH on unclear diet and their agreement with DIF, but they did not find a degree of agreement between serological tests and DIF. Hadjivassilou et al. [25] examined the prevalence of TG6 in a cohort of patients with DH, and they found that TG6 antibodies appear to develop more frequently in patients where tolerance to gluten was broken. Jaskowski et al. [29] assess the performance of a novel combined antigen-screening assay for gluten sensitivity. This study showed that the new IgA/IgG anti-tTG/DGP EIA screen was slightly more sensitive than IgA anti-tTG alone in pediatric CD. Kasperkiewicz et al. [27] explored the anti-GAF3X ELISA and compared it with a panel of classic CD-related serologic tests in patients with DH, hence the novel anti-GAF3X ELISA showed a higher sensitivity to detect CD-associated autoantibodies. Marietta et al.26 found an association between intestinal damage and the expression of IgA-tTG and IgA-eTG in patients with DH. McCord and Hall III [33] found the presence of IgA antireticulin and antiendomysium in both the serum and gut secretions of DH patients, suggesting that these antibodies arise as a result of the mucosal immune response and that IgA of mucosal origin can persist in the serum of patients with DH. Natynki et al. [34] investigated whether patients with DH and CD have circulating autoantibodies against the BP180 autoantigen. Their results did not exclude the possibility that intermolecular epitope spreading could explain the switch from DH to bullous pemphigoid in elderly patients. Sardy et al. [34] found that eTG, rather than tTG, is the dominant autoantigen in DH and explains why skin symptoms appear in a proportion of patients with gluten sensitive disease. Sugai et al. [13] showed that the detection of GDP antibodies was the most reliable tool in identifying gluten sensitivity in DH patients presenting a wide range of intestinal damage. Finally, Velikova et al. [35] explored the performance of a panel of CD-related antibodies in patients with DH, and found that these serologic tests could be used with advantages in the diagnostic process of patients with DH. Furthermore, DH patients who are positive for the serologic parameters investigated could have routine monitoring for gastrointestinal complications typical of gluten-sensitive enteropathy.

## Discussion

This systematic review and meta-analysis evaluated the diagnostic accuracy of serological tests for DH, including IIF for IgA EMA with ELISA for IgA tTG and ELISA for IgA eTG, using DIF as the reference standard. The objective was to compare the performance of these tests and understand their clinical applicability in diagnosing DH.

### Diagnostic strategies

The S2k guidelines initiated by the European Academy of Dermatology and Venereology (EADV) highlight DIF as essential for detecting granular IgA deposits in the dermal papillae of the perilesional skin [36]. Our results are consistent with these guidelines, confirming the high specificity and sensitivity of DIF, supporting its use as a primary diagnostic tool supplemented with serological tests.

Similarly, van Beek et al. [37] emphasise the importance of DIF in diagnosing autoimmune blistering disorders (AIBDs), including DH. They advocate for DIF as a primary diagnostic tool, supported by serological tests, which our study also highlights as essential to confirm the diagnosis of DH. Our meta-analysis highlights notable differences in sensitivity and specificity across diagnostic tests. For instance, the low specificity of IIF and ELISA-tTG in 'Sample at diagnosis not-GFD' scenarios suggests that these tests are better suited as initial screening tools rather than standalone diagnostics. Combining serological tests with DIF could improve diagnostic accuracy, especially in complex cases where clinical suspicion remains high. The immunological overlap between DH and CeD reinforces the importance of combining serological and histopathological methods for an accurate diagnosis [39]. Although DIF remains the gold standard for detecting granular IgA deposits in dermal papillae, serological tests such as TG3 and TG2 antibodies serve as essential complementary tools. TG3 autoantibodies, specific to DH, are particularly valuable for confirming the diagnosis, especially in patients without classical gastrointestinal symptoms [3, 39]. However, their diagnostic sensitivity decreases in patients adhering to a GFD, highlighting the need for customized diagnostic strategies that integrate the nutritional context with serological and histopathological findings.

### Serological tests

Our findings emphasise the importance of selecting the appropriate diagnostic test based on the clinical context. IIF for IgA EMA and ELISA for IgA tTG show high sensitivity but lower specificity in patients with cutaneus lesions suspicious for DH on a gluten containing diet, making them suitable for confirming the diagnosis. The ELISA for IgA eTG, with its more balanced sensitivity and specificity, is not a good diagnostic test for DH. These results underscore the concept that, in patients with cutaneous lesions suspicious of DH, the same diagnostic approach as used for patients with CD without cutaneous lesions should be adopted: IgA transglutaminase (TG) evaluation followed by duodenal biopsies to assess the presence of histological damage [38].

The meta-analysis by Elwenspoek et al. [39] identified a strong diagnostic value in patients with conditions associated with CD, such as DH, highlighting the importance of targeted testing. Our results support this by demonstrating the efficacy of serological tests in diagnosing DH.

The meta-analyses found some variations in sensitivity and specificity for the serological tests analyzed in the different scenarios. The comparison between 'Sample at diagnosis not-GFD' and 'Sample at follow-up GFD' revealed notable differences in sensitivity and specificity. Our findings confirm the well-established understanding in clinical practice that a gluten-free diet reduces the sensitivity of serological tests such as ELISA for IgA tTG and IIF for IgA EMA [7, 8]. This reduction in sensitivity highlights the importance of considering the dietary status when interpreting these test results, which is a standard practice among clinicians. These results were also obtained by Ciacci et al. [7] and Sugai et al. [8], when they studied the expression of serologic IgA in celiac patients who adhere to GFD. Furthermore, in "Sample DH in follow-up patients with FGD and SD or healthy subjects" the IIF tests for IgA EMA and ELISA for IgA tTG had a high specificity, suggesting that it is a good tool to exclude DH diagnosis in this scenario.

### Strenghts and limitations

This analysis is subject to certain limitations, including significant variability between studies and a low number of studies included in the meta-analysis, which has resulted in high CrIs for the results. Furthermore, the reliance on DIF as the reference standard as the reference standard introduces additional challenges, such as variability in biopsy quality, operator dependence, and lack of universal standardization, which could affect diagnostic accuracy. To address the limitation of sparse data, we have aggregated IIF and ELISA-tTG results because they had similar accuracy trends, enhancing the robustness of the analysis. However, a key strength is the use of Bayesian methods that account for these imperfections, offering a more accurate assessment of the diagnostic accuracy of serological tests for DH.

Assessment of risk of bias revealed significant concerns in patient selection and index test domains. The wide variability in test specificity, particularly for ELISA-tTG and IIF, raises questions about standardization in study methodologies and population selection. This heterogeneity may reflect differences in cutoff definitions, patient characteristics, or test protocols between studies. High risk was particularly noted in several studies, indicating potential issues with representativeness and test application or interpretation. These concerns can affect the validity and reliability of the diagnostic tests that are being evaluated. The results should be interpreted with caution, considering the heterogeneity of the study designs, patient populations, and test methodologies in the included studies.

## Conclusions

The findings underscore the importance of selecting diagnostic tests based on clinical context. The observed variability in test performance, particularly concerning patient diet, highlights the need for an advanced approach to diagnostic testing, incorporating multiple serological tests to improve accuracy. For instance, positive transglutaminase serological tests may eliminate the need for extensive dermatological evaluation, as a perilesional biopsy with DIF could suffice to confirm the diagnosis and initiate a gluten-free diet. However, in cases of dermatitis with minimal histological damage, DIF remains essential to clarify the diagnosis and avoid mismanagement. Specifically, the eTG ELISA test demonstrates poor diagnostic accuracy, making its use inefficient and financially unjustified. On the other hand, tTG ELISA and EMA tests exhibit high sensitivity in patients on a gluten-containing diet and high specificity when distinguishing confirmed DH cases from patients with other skin lesions or healthy individuals. These characteristics make tTG ELISA and EMA valuable tools for confirming or ruling out DH in appropriate clinical settings, ultimately allowing for more targeted and cost-effective diagnostic strategies.

Future research should focus on standardising study designs and improving patient selection methods to enhance the reliability of diagnostic accuracy studies, while exploring strategies that integrate serological and histological assessments to streamline the diagnostic process.

## Supplementary Information

Additional file 1.

## Data Availability

Data sharing is not applicable to this article as no datasets were generated or analyzed during the current study. All data supporting the findings of this review are available in the included studies and referenced publications.

## Abbreviations

DH: Dermatitis herpetiformis
CeD: Celiac disease
DIF: Direct immunofluorescence
IIF: Indirect immunofluroescence
ELISA: Enzyme-linked immune sorbent assay
EMA: Endomysial antibodies
tTG: Tissue transglutaminase
eTG: Epidermal transglutaminase
IB: Immunoblotting
EIA: Enzyme Immunoassay
IgG: Immunoglobuline G
IgA: Immunoglobuline A
GFD: Gluten-free diet
